# Practical Approaches to Treat ED in PDE5i Nonresponders

**DOI:** 10.14336/AD.2019.1028

**Published:** 2020-10-01

**Authors:** Zhonglin Cai, Xiaoqing Song, Jianzhong Zhang, Bin Yang, Hongjun Li

**Affiliations:** ^1^Department of Urology, Peking Union Medical College Hospital, Peking Union Medical College, Chinese Academy of Medical Sciences, Beijing, China; ^2^Department of Pathology, First Affiliated Hospital and College of Basic Medical Sciences, China Medical University, Shenyang, China.; ^3^Department of Urology, The Affiliated Hospital of Qingdao University, Qingdao, China

**Keywords:** erectile dysfunction, phosphodiesterase type 5 inhibitors, rescue strategy, nonresponders

## Abstract

Erectile dysfunction (ED) is a common sexual disorder in adult males and one of the most important factors affecting their quality of life and that of their partners. Although PDE5 inhibitors (PDE5is) are the first choice for improving erectile function, there is a substantial proportion of ED patients, termed PDE5i nonresponders, who do not respond to PDE5is. Because of the lack of effective therapies, these patients always have serious social and psychological problems due to ED, which should be addressed. Here, we review the available literature about ED and PDE5is and propose several strategies for mitigating ED in PDE5i nonresponders.

## 1. Introduction

Erectile dysfunction (ED) is a common sexual disorder in adult males and has significant biological, psychological and social effects on the quality of life (QOL) of men with ED and their sexual partners or spouses [1-2]. Indeed, the high prevalence of ED has become a global health concern [3]. The percentages of men affected by ED are as follows: 14.3-70% of men aged ≥60 years, 6.7-48% of men aged ≥70 years, and 38% of men aged ≥80 years [4]. Many treatments are available for ED, including psychotherapy, oral medication, vacuum constriction devices (VCDs), intraurethral drugs, intracavernous drugs, and implantable prosthetics. Among them, phosphodiesterase type 5 inhibitors (PDE5is), as a first-line treatment, are recognized as the most satisfying and effective drugs for ED [5-6]. However, with the increasing administration of PDE5is in clinical practice, it has found that approximately 30-35% of ED patients cannot respond to PDE5is [7]. Although the unreasonable use of PDE5is, including the use of inadequate dosages, administration of a single dose, and lack of dosage regulation, is considered the main reason for the lack of a response to PDE5is [8], 50-70% of ED patients still do not respond to PDE5is after restoring rational PDE5i use [9]. In general, the detailed mechanisms of the lack of a response to PDE5is in ED remain to be clarified, and treating these patients is a challenge that urgently needs to be addressed.

**Table 1:** Direct evidence of rescue treatment in PDE5i nonresponders.

Approaches for rescue treatment in PDE5i nonresponders	PDE5i nonresponse				Rescue therapy with/without PDE5is				Ref.
	PDE5i	Dosage	Dosing regimen	PDE5i	Dosage	Dosing regimen	Other therapeutic approaches	Salvage success rate	
Increased PDE5i doses	Sildenafil	100 mg	On demand	Sildenafil	150 or 200 mg	On demand	NA	24.1%	38
Different dosing regimens	Tadalafil	20 mg	On demand	Tadalafil	Flexible doses of 10 and 20 mg	Once daily	NA	58.0%	46
Different dosing regimens	Vardenafil	20 mg	On demand	Vardenafil	10 mg	Once daily	NA	38.8%	47
Different PDE5is	Sildenafil	100 mg	On demand	Vardenafil	Flexible doses of 10 and 20 mg	On demand	NA	12.0%	48
Different PDE5is	Sildenafil	≤100 mg	On demand	Vardenafil	Flexible doses of 5, 10 and 20 mg	On demand	NA	53.0%	49
Non-drug therapeutic approaches with or without PDE5is	At least one PDE5i (20 mg for tadalafil or vardenafil hydrochloride, 100 mg for sildenafil)			Taking the same PDE5i as PDE5i nonresponse			VED	70.0%	59
Non-drug therapeutic approaches with or without PDE5is	Tadalafil	5?mg	Once daily	NA	NA	NA	LiSWT	41.7%	62
Non-drug therapeutic approaches with or without PDE5is	PDE5is (unclear in detail)			NA	NA	NA	LiSWT	60.0%	63
Non-drug therapeutic approaches with or without PDE5is	Sildenafil (100 mg), tadalafil (20 mg), and vardenafil (20 mg) as needed or tadalafil (5 mg) daily			Taking the same PDE5i as PDE5i nonresponse			LiSWT	67.3%	64
Attention to psychological factors	Sildenafil	100 mg	On demand	Sildenafil	100 mg	On demand	Trazodone (50 or 100 mg, once daily)	66.7%	68
PDE5is combined with other non-PDE5i drugs	Highest available dosage of sildenafil, tadalafil, or vardenafil therapy			Tadalafil	10 mg	Once daily	Testogel (5 g up to 10 g, once daily)	33.1%	110
PDE5is combined with other non-PDE5i drugs	Sildenafil	100 mg	On demand	Sildenafil	100 mg	On demand	Oral testosterone undecanoate (Restandol, 80 mg, bid or tid)	34.3% after testosterone replacement only, 37.5% more after combined therapy	111
PDE5is combined with other non-PDE5i drugs	Tadalafil	20 mg	On demand	Tadalafil	20 mg	Twice a week	Testogel (5 g, once daily)	NA (improvement in IIEF-EF)	113
PDE5is combined with other non-PDE5i drugs	Sildenafil	100 mg	On demand	Sildenafil	100 mg	On demand	Atorvastatin (40 mg, once daily)	NA (significant improvements in all IIEF-5 questions and GEQ)	117

Salvage success is defined as being able to successfully complete sexual intercourse. NA, not applicable; VED, vacuum erectile device; LiSWT, low-intensity shockwave therapy; IIEF, international index of erectile function; EF, erectile function; GEQ, global efficacy question.

ED is closely related to aging. Penile erection is a complex physiological activity involving the neuroendocrine vascular tissue system [10], and aging can not only cause tissue dysfunction related to this physiological activity, including dysfunction involving the nerves, blood vessels, cavernous tissue and reproductive hormones, but also increase the risk of penile ED [11]. Aging also increases the risk of various chronic diseases in the elderly, such as cardiovascular diseases, diabetes, metabolic syndrome, late-onset hypogonadism (LOH), and lower urinary tract symptoms (LUTSs) [11]. These chronic diseases have been recognized as risk factors for ED and can cause and/or aggravate ED [12]. It has been shown that the prevalence of ED ranges from 1-10% for men aged under 40 years, 2-15% for men aged 40-49 years, 22-31% for men aged 50-69 years, 20-40% for men aged 60-69 years, and 50-100% for men over 70 years old [13]. Therefore, aging is an important risk factor for ED development. At the same time, the changes caused by aging will also increase the difficulty of ED treatment, which is an important factor of the lack of a response to PDE5i treatment. It is worth noting that according to previous reports, approximately 15% of the global population will be over 65 years old by the year 2025 due to the increase in life expectancy [11, 14]. Therefore, sexual dysfunction, especially ED, in elderly men is a problem that cannot be ignored. Moreover, PDE5i nonresponsiveness in older men as a result of age is a major challenge that urgently needs to be addressed. Here, we propose several strategies for treating ED in PDE5i nonresponders and present a summary of the direct evidence of rescue treatment in PDE5i responders in Table 1.

## 2. Possible causes for ED in PDE5i nonresponders

PDE5i-nonresponsive ED patients always have comorbidities, including diabetes, cardiovascular disease, and metabolic syndrome, among others [15-16]. The nerves, blood vessels, and cavernous tissues in the penis can be injured as a result of these comorbidities, which is the basis of ED development [17-18]. Although PDE5is can improve erectile function through the cGMP-PKG and cAMP-PKA signaling pathways, among others, the therapeutic effect of PDE5is depends on the proper function of tissue effectors, such as nerves, blood vessels and cavernous tissues. Therefore, the above comorbidities affect the therapeutic efficacy of PDE5is in ED by damaging these tissues in the penis. As diabetes can disrupt the balance of sex hormones, such as by lowering testosterone levels to affect erection function [19-20], the presence of such comorbidities may be an important cause of PDE5i nonresponsiveness. In addition, the side effects of some drugs used for comorbidities, such as selective serotonin-reuptake inhibitors, thiazides, and β-blockers, affect erectile function and thereby weaken the effect of PDE5is on ED [21-22]. Interestingly, unhealthy behaviors, such as excessive drinking, smoking, and a high-fat diet, can also promote the development and progression of these comorbidities in men, in turn affecting the efficacy of PDE5is in the treatment of ED [23-25]. Hence, lifestyle factors should be emphasized in ED treatment when there is the lack of a response to PDE5is.

In addition to comorbidities, complications from treatments for diseases are common reasons for ED. For example, radical prostatectomy (RP) can cause cavernous nerve injury and vascular damage, and pelvic radiation therapy for prostate cancer can result in the death or fibrosis of cavernosal smooth muscle cells, nerve cells, and vascular smooth muscle cells, among others [26-27]. These complications destroy the tissues that are the basis for the therapeutic efficacy of PDE5is in ED, thus reducing the efficacy of the drug. If the damage is significant enough, the ED patient will not respond to PDE5i therapy. Furthermore, spinal cord injury can directly damage nerves, resulting in damage to or the attenuation of sexual activity-related nerve reflex arcs, e.g., nerves and blood vessels in the penis, by altering neuroendocrine reflexes [21, 28]. Such traumas involving injury to nerves, blood vessels, and cavernous tissues related to erectile function can cause the abovementioned adverse events.

It has been found that nonresponsiveness to sildenafil for ED is related to incorrect administration factors, such as taking the medication after heavy meals, a lack of sexual stimulation, administration timing, too few attempts and other reasons [22, 29]. Additional mechanisms are unknown. However, correcting PDE5i administration or replacing the PDE5i regimen according to the metabolic and distribution characteristics of different PDE5i drugs can attenuate the abovementioned problems associated with incorrect drug administration.

Male erectile function is closely related to psychological factors [30]. Mental disorders can cause or aggravate ED, and such disorders can be derived from society, family, spouse, and self [30-31]. During PDE5i treatment for ED, the sexual psychological disorders of ED patients will directly influence the effects of PDE5i treatment for ED and could even lead to the lack of a response to the treatment [31-32]. Fortunately, psychological interventions, such as cognitive behavioral therapy and sexual counseling, can improve the patient’s erectile function or enhance the efficacy of PDE5is in the treatment of ED [31-32]. Therefore, psychological factors may be another cause of PDE5i nonresponsiveness.

## 3. Approaches to improve ED in PDE5i nonresponders

### 3.1 Improved pharmacotherapy with PDE5is

#### 3.1.1 Sufficient medication attempts

During treatment with an on-demand regimen, insufficient medication attempts is a common reason for patients not achieving a response to PDE5is [29], and the reason may be associated with an insufficient blood concentration. It has been shown that the coital success rate was elevated with increased sildenafil frequency but remained stable after eight doses (with a success rate of up to 86%) [33]. For vardenafil, another short-acting drug, research has also indicated that the likelihood of successful penetration and intercourse increases with additional medication attempts, with the effect of vardenafil on ED reaching a plateau at approximately the fourth dose [34]. Therefore, it has been suggested that patients who fail to achieve treatment success initially should continue treatment with increased doses [34]. Interestingly, for the long-acting PDE5i drug tadalafil (as needed), a substantial proportion of ED patients who fail on the first attempt will achieve success provided the treatment is maintained [35]. Moreover, approximately 80% of patients achieved successful sexual intercourse within eight attempts at 10 mg and within four attempts at 20 mg, which also indicates that fewer attempts are required with a greater dose; these findings reflect the importance of an adequate PDE5i blood level in the treatment of ED [35]. Overall, to ensure efficacy, ED patients should be given regular and sufficient PDE5i doses. Indeed, sufficient medication attempts is an important strategy for improving the therapeutic efficacy of PDE5is in nonresponders.

#### 3.1.2 Increased PDE5i doses

The therapeutic efficacy of PDE5is in ED increases with increasing dose. ED patients treated with 50 mg of sildenafil on demand achieved 1.4 sexual intercourse attempts per week, with a success rate of 82% [36]. However, with 100 mg of sildenafil as needed, the number of sexual intercourses attempts per week increased to 1.7, with a 91% success rate, indicating that increasing the dose of sildenafil can increase both the number of sexual intercourse attempts and the sexual success rate [36]. Early sildenafil treatment failure does not necessarily mean that there will be no effect in the future, and on-demand administration can be increased to 100 mg to increase the success rate of treatment, further demonstrating that increasing the dose can improve the effect of sildenafil on ED [37]. Additionally, the dose of sildenafil can be increased to 200 mg, which can render 24.1% of sildenafil nonresponders responsive to the drug [38]. Although 200 mg of sildenafil on demand has a higher incidence of adverse effects, increasing the administered dose within a safe range is a viable treatment strategy in PDE5i nonresponders. Vardenafil has a similar dose-effect trend as sildenafil. Clinical studies on vardenafil have indicated that regarding indexes of the therapeutic efficacy of PDE5is in ED, such as the Sexual Encounter Profile question 2 (SEP-2) (penetration) and SEP-3 (maintenance) scores, and overall satisfaction with the sexual experience, first-attempt and subsequent success rates increase with increasing dose [39-40]. For long-acting PDE5is, such as tadalafil, increasing clinical studies have also demonstrated gradual increases in indexes of erectile function, such as the International Index of Erectile Function (IIEF)-EF, SEP-2, SEP-3, and Global Assessment Question (GAQ) scores, with increasing dose, regardless of the dosing regimens, e.g., as needed or daily [41-43]. Additionally, no association with the severity of ED has been observed in the gradually enhanced therapeutic effect with increasing dose [44], suggesting that an increased PDE5i dose may be key for improving efficacy in ED treatment. Furthermore, during early treatment, PDE5i nonresponders may not actually be nonresponders, and it is recommended to use PDE5is starting from the highest label-approved dose followed by down-titration to minimize the frustration caused by inadequate response due to inadequate drug dosage, thereby reducing the impact of psychological factors on the treatment [12, 45]. Thus, increased doses may be an important strategy in PDE5i nonresponders.

#### 3.1.3 Different dosing regimens

The use of PDE5is for ED has two dosing regimens, i.e., on demand and daily. In ED patients who were previously unresponsive to on-demand tadalafil, daily flexible tadalafil (10/20 mg) significantly improved the IIEF-EF and SEP-3 scores compared to baseline and on-demand tadalafil, and more patients had achieved an erection at the end point [46]. Thus, tadalafil with a daily dosing regimen (10/20 mg) is considered an effective salvage treatment in previous on-demand tadalafil nonresponders [46]. Regarding the short-acting PDE5i vardenafil, one study on daily dosing for on-demand nonresponders has shown that according to the SEP-3 score, 38.8% of patients with ED who previously did not respond to the as-needed regimen became responders; the study suggests that daily vardenafil can be applied as a salvage strategy in ED patients with no response to the on-demand regimen [47]. In summary, regardless of whether a PDE5i is short acting or long acting, the daily dosing regimen allows ED patients who do not respond to the on-demand dosing regimen to achieve appropriate blood levels for improved therapeutic efficacy and is thus expected to be an important strategy for treating on-demand regimen nonresponders.

#### 3.1.4 Different PDE5is

Because of the heterogeneity among individuals, each ED patient’s response to PDE5is differs. The advantage of individual responsiveness to different PDE5is may be an important strategy for improving the efficacy of PDE5is in nonresponders.

Vardenafil can be used for salvage treatment in a small proportion of sildenafil (100 mg) nonresponders [48], and flexible-dose vardenafil (5-20 mg) for moderate to severe ED in sildenafil nonresponders achieved high insertion and sexual success rates [39, 49]. Most patients receiving vardenafil reported improved erections and the achievement of normal erections [42,49]. Therefore, vardenafil can be used to treat ED patients who do not respond to sildenafil. As a long-acting PDE5i, tadalafil has been shown in several studies to change the dosing and sexual attempt behavior of ED patients, reflecting the extended period of efficacy of tadalafil [50]. Compared to sildenafil, as-needed tadalafil has several advantages, including a broader window of opportunity available for sexual activity, decreased time concerns, reduced IIEF-EF scores, reduced side-effect severity scores, more satisfying erection hardness and more sexual attempts [51-52]. Therefore, more patients prefer on-demand tadalafil [52]. In conclusion, changes in the dosing and sexual attempt behavior in patients taking tadalafil on demand and patient preferences for tadalafil suggest that on-demand tadalafil may be superior to sildenafil and an important strategy for treating PDE5i nonresponders. Unfortunately, in ED patients who do not respond to the maximum dose of sildenafil or vardenafil, the recommended maximum dose of tadalafil (20 mg) did not significantly improve the IIEF-5, SEP-2, SEP-3, or GAQ score by the end point [53]. However, it has been shown that daily tadalafil can serve as a rescue therapy in previous on-demand tadalafil nonresponders [46]. Daily tadalafil may have potential as a salvage treatment in ED patients nonresponsive to sildenafil or vardenafil, but more studies are needed to demonstrate this hypothesis. It is worth mentioning that first-generation PDE5is had a high discontinuation rate due to side effects. According to the results of a meta-analysis, avanafil, a new PDE5i, showed efficacy comparable to that of first-generation PDE5is but a lower incidence of side effects and thus a reduced discontinuation rate [54]. By increasing compliance, reducing the discontinuation rate is beneficial to ensure the smooth implementation of related treatment approaches in PDE5i nonresponders. Nonetheless, research to date on avanafil for the treatment of PDE5i nonresponders is insufficient, and further studies are needed. Overall, other PDE5is can be used in ED patients who do not respond to a given PDE5i.

#### 3.1.5 Combined use of long-acting and short-acting PDE5is

PDE5i nonresponders always suffer from pathological changes in penile vascular tissue. Most ED patients with no response to 100 mg of sildenafil have been found to also have vascular diseases, such as arterial insufficiency, mixed vascular insufficiency, and cavernous venous occlusive disease [48]. The chronic use of low-dose, long-acting PDE5is, such as tadalafil, contributes to repair of the injury to penile vascular endothelial cells, and short-acting PDE5i administration on demand has an instantaneous effect on improving erection [55]. Thus, the combination of long-acting and short-acting PDE5is may be an important direction for treating PDE5i non-responders. Compared with 5 mg of tadalafil daily alone, 5 mg of tadalafil daily combined with sildenafil on demand resulted in higher IIEF-5 scores and percentages of 'yes' responses to SEP-4 and SEP-5 at the end of a 12-week follow-up period. Interestingly, in the combination group, the IIEF-5 scores of severe ED patients were significantly higher [56]. The side effects were similar in the two groups. Overall, the long-term, low-dose administration of long-acting PDE5is combined with short-acting PDE5is on demand is an important strategy in those who do not respond to PDE5i treatment.

### 3.2 Non-drug therapeutic approaches with or without PDE5is

Vacuum erectile devices are a noninvasive treatment for ED, and their first treatment efficiency can reach 90% [57]. For ED patients who are not satisfied with the therapeutic effect of PDE5is, the combination of a PDE5i and a vacuum erectile device can improve the IIEF-EF score, with a good therapeutic effect [58]. For example, a PDE5i combined with a vacuum erectile device significantly improved the SEP-2, SEP-3, and global patient assessment scale (GPAS) scores in PDE5i nonresponders. The IIEF-5 score increased from 9 to 17.6, and there were no obvious side effects [59]. Accordingly, a PDE5i combined with a vacuum erectile device can be considered an important strategy for treating PDE5i nonresponders.

Low-intensity shock-wave therapy (LISWT) is a new treatment method for ED. The low-intensity shock wave can release endogenous media and nitric oxide (NO) to promote the regeneration of neovascularization and improve both the blood supply to the cavernous artery and vasodilation [60]. A meta-analysis of LISWT for ED has shown that the effect of LISWT on improving ED can last for 6 and 12 months [61]. For example, LISWT was effective for 41.7% of ED patients who did not respond to PDE5is, resulting in satisfactory erectile function [62]. Similarly, in a study on LISWT for PDE5i nonresponders, it was confirmed that LISWT was effective for approximately 60%, and the therapeutic effect continued until the end of the 12-month follow-up period [63]. A PDE5i [sildenafil (100 mg), tadalafil (20 mg), or vardenafil (20 mg) with a trial of at least four attempts, or tadalafil (5 mg) with a trial of at least 28 days] combined with LISWT (twice a week for 12 weeks) also resulted in 67.3% of PDE5i nonresponders achieving successful sexual intercourse [64]. In summary, the administration of LISWT with or without a PDE5i is an important direction in treating PDE5i nonresponders.

### 3.3 Attention to psychological factors

ED is a complex disease, and minimal organic ED can induce psychogenic components. The incidence of psychological distress, including anxiety, depression and stress, is also closely related to the severity of ED [65-66]. Therefore, psychological distress is a crucial factor affecting the therapeutic effect on ED. It has been shown that psychological intervention combined with PDE5is could restore erectile function more effectively than psychological intervention alone, as based on the IIEF score [67]. Drugs are considered an adjunct to the treatment of psychosocial disorders, and sildenafil combined with trazodone, an antidepressant, was effective for men with ED who initially failed to respond to oral sildenafil and allowed them to achieve satisfactory sexual activity [68].

As one type of psychological intervention, sexual counseling can not only improve erectile function and sexual satisfaction but also increase treatment compliance in patients with ED caused by RP and penile prosthesis implantation, extended device application or hypospadias repair performed in childhood [69-73]. A meta-analysis has demonstrated that taking PDE5is to improve erectile function requires other comprehensive management, including sexual counseling, exercise and lifestyle changes [74]. While an ED patient is using a PDE5i, the addition of counseling therapy can more effectively improve sexual function and sexual satisfaction, even in the elderly male population [74].

Cognitive behavioral therapy is another psychotherapeutic approach that can be applied to treat psychogenic ED. Compared to sildenafil or cognitive behavioral therapy alone, the combination of cognitive behavioral therapy and sildenafil significantly improved the IIEF-EF score, with a higher success rate for treating ED within the first 4 weeks of therapy [75]. At the same time, PDE5is combined with cognitive behavioral therapy can both effectively improve ED and alleviate anxiety, depression and other mental problems, and the effect can be maintained for 15-18 months compared with PDE5is alone [31, 76].

Increased satisfaction is related to increased therapeutic efficacy, partner support, and the number of sexual attempts [77]. In fact, the partner’s role in ED treatment is an important influential and psychological factor that can be presented in many different ways, such as partner-initiated communication to relieve the anxiety and stress of ED patients, partner-facilitated activities to improve intimacy, partner-facilitated adaptation of sexual techniques and lifestyle changes, and partner-encouraged professional counseling and follow-up of medical treatment [3]. Thirty-four percent of ED patients express willingness to visit a doctor if their sexual partners asked them to do so [3], and 10.7% of ED patients cited their partner’s preference as an influential factor of their treatment choice [78]. Partner negativity is also an important factor that affects the responsiveness of ED patients to PDE5is [29]. Therefore, the roles of partners should be considered when treating PDE5i nonresponders. In summary, paying attention to psychological factors is important for enhancing the effects of PDE5is on ED, even in nonresponders.

### 3.4 Focus on the comorbidities of ED

#### 3.4.1 Selection of PDE5is with greater effects on ED

Many diseases are likely associated with ED, causing refractory ED and a poor prognosis. Males who have metabolic syndrome, diabetes, hypertension, coronary heart disease, and hypogonadism, among other conditions, are particularly susceptible to ED [79]. Moreover, the comorbidities of ED are reasons why patients with ED might not respond to PDE5is, although the potential mechanisms differ. Thus, the selection of a PDE5i that is suitable or more suitable for the treatment of ED along with any comorbidities is a key strategy for improving the therapeutic efficacy in patients with a poor or no response to some PDE5is.

##### 3.4.1.1 PDE5is, ED and premature ejaculation

In some ED patients, premature ejaculation is associated with ED, forming a vicious cycle [80]. It has been reported that 29.5% and 52.4% of patients with mild and severe ED, respectively, experience premature ejaculation [81]. To conceal the decrease in hardness during sexual intercourse, some ED patients often subjectively and consciously hope to end sexual intercourse as soon as possible (ejaculation is an important sign of the end of sexual intercourse), resulting in premature ejaculation [82]. The poor sexual performance caused by premature ejaculation will further aggravate the anxiety and pain of patients and lead to reactive ED, thus weakening the therapeutic effect, including the response to PDE5i treatment [82]. In addition, the treatment of premature ejaculation, whether psychologically or medically, aims to reduce the intensity of local stimulation and sexual excitation to delay ejaculation, and a reduction in stimulation intensity and sexual excitation leads to or aggravates ED, further the weakening therapeutic effect [83]. Hence, premature ejaculation is an important comorbidity of ED, and ED along with premature ejaculation is a reason for the lack of a response to PDE5i treatment. Interestingly, PDE5is can reduce sympathetic nerve tension in the vas deferens and seminal vesicles through the NO/cGMP signaling pathway and relax smooth muscle to achieve the dual goals of treating ED and delaying ejaculation [84]. Several studies have confirmed that regardless of whether they are combined with drugs for treating premature ejaculation, such as paroxetine (20 mg daily) and fluoxetine (90 mg once a week), PDE5is, including sildenafil (25-100 mg on demand), vardenafil (10 mg on demand) and tadalafil (20 mg on demand), can enhance ejaculation control, effectively prolonging the ejaculation time, improving anxiety and overall satisfaction and reducing the recovery time after orgasm to achieve a second erection [85-89]. Although a few studies have indicated that PDE5is cannot prolong the ejaculation time, PDE5is can help improve anxiety and overall satisfaction, which can partly alleviate the vicious cycle of ED and premature ejaculation from a therapeutic perspective [90]. Furthermore, the emotional outcomes of the loss of erectile function are more painful than those of premature ejaculation. Although PDE5is improve erectile function and enhance erectile confidence, patients with ED and premature ejaculation can achieve delayed ejaculation without the fear of losing erectile stiffness, but the ability to delay ejaculation often does not exceed that before ED [82]. Therefore, drugs for premature ejaculation, as well as psychosocial and behavioral therapy, are needed to increase the ejaculation threshold from a biological point of view and enable patients to achieve the physical and psychological changes necessary for delayed ejaculation [82, 91]. Premature ejaculation will aggravate the difficulty using PDE5is for the treatment of ED. PDE5is, including sildenafil, tadalafil and vardenafil, can be used to treat ED; however, both drugs for treating premature ejaculation and psychosocial behavioral therapy should be implemented to break the vicious cycle of ED and premature ejaculation, restore responsiveness to PDE5is and improve overall life satisfaction in the treatment of ED.

##### 3.4.1.2 PDE5is, ED and diabetes

The presence of diabetes increases the difficulty of treating ED. In patients with type 1/2 diabetes and ED, as-needed tadalafil remarkably improved the IIEF-EF, SEP-2 and SEP-3 scores; thus, it is believed that tadalafil (10 mg or 20 mg as needed or 20 mg three times per week) can enhance erectile function in men with diabetes and ED [92-94]. Additionally, flexible-dose vardenafil (5-20 mg) in men with type 1 diabetes and ED significantly improved the mean success rate according to the SEP-2 and SEP-3 scores [95]. Nonetheless, the effect of sildenafil on ED in patients with diabetes was moderate, and the response rate was relatively low [93]. Accordingly, tadalafil or vardenafil may be a suitable PDE5i for ED patients with diabetes.

##### 3.4.1.3 PDE5is, ED and spinal injury

Spinal injuries are closely related to the therapeutic efficacy of PDE5is in secondary ED. Sildenafil, tadalafil, and vardenafil are effective for ED secondary to spinal injury, at a rate of 85%, 72% and 74%, respectively [96-97]. Interestingly, upper motor neuron (UMN) lesions are associated with PDE5i treatment success, and poorer responses to PDE5is are observed in patients with lower motor neuron lesions and horsetail injuries [97]. The efficacy of sildenafil in ED patients with UMN lesions was significantly higher than that of the placebo (85% vs 25%, P<0.05) [98]; only 28% of patients with non-UMN lesions showed a response to sildenafil, although the difference was not significant [98]. Furthermore, research has shown that compared to sildenafil, tadalafil allowed significantly more ED patients to achieve normal sexual function up to 24 h postdosing, as well as a more satisfying sex life [99]. In other words, the effect of PDE5is on ED secondary to spinal injury is limited to the spinal injury site. Although sildenafil, tadalafil, and vardenafil are effective for ED secondary to UMN lesions, their suitability is not clear and requires further study.

##### 3.4.1.4 PDE5is, ED and RP

One common complication after RP is ED. The therapeutic effect of PDE5is on ED is closely associated with the degree of neurovascular sparing. In one study, 76% of patients with bilateral nerve-sparing treatment and 53.5% of patients with unilateral nerve-sparing treatment responded to sildenafil, whereas only 14.2% of patients with non-nerve-sparing treatment responded to sildenafil [100]. Similar to sildenafil, 10 mg and 20 mg of vardenafil as needed for ED secondary to nerve-sparing RP improved erectile function according to the IIEF domains and allowed patients to have more satisfying sexual experiences [100]. Interestingly, a systematic review has shown that after bilateral nerve-sparing RP, avanafil on demand was the most effective PDE5i for the recovery of drug-assisted erectile function; tadalafil was equally effective when used on demand and daily, although vardenafil significantly improved drug-assisted erectile function recovery only when used on demand [101]. In addition to the degree of neurovascular bundle preservation, the effect of sildenafil on ED has been shown to correlate with age, the preoperative erectile function status, and the interval before starting treatment [102-103]. Therefore, when PDE5is are used to treat ED after RP, the therapeutic effect depends on more than the PDE5i, and avanafil, tadalafil, sildenafil and vardenafil are all candidates for treating ED after RP with neurovascular bundle preservation.

##### 3.4.1.5 PDE5is, ED and other comorbidities

Dyslipidemia is a common etiological or causative factor of ED. The effectiveness of vardenafil was not affected by the TC/HDL-C ratio, the LDL-C level, or the presence of metabolic syndrome, and it has been recommended for treating ED in patients with dyslipidemia [104]. ED patients with comorbid benign prostate hyperplasia/lower urinary tract symptoms (BPH/LUTSs) can benefit from the long-term use of long-acting tadalafil [105]. Therefore, it is necessary to consider the characteristics of the comorbidities during ED treatment with PDE5is, and more suitable PDE5is should be selected for treating ED. Fortunately, an integrated analysis of 11 double-blind, placebo-controlled studies showed that on-demand tadalafil (10 mg or 20 mg) significantly improved the IIEF-EF, SEP-3 and GAQ scores of ED patients with different comorbidities, such as diabetes mellitus, hypertension, cardiovascular disease, hyperlipidemia, depression, and BPH [79]. Therefore, it is believed that tadalafil, a long-acting PDE5i, can significantly improve the erectile function of patients with ED and different comorbidities. Tadalafil may be widely suitable for ED in most patients with comorbidities, but further research is needed.

#### 3.4.2 Management of medications for comorbidities of ED

The comorbidities of ED include diabetes, cardiovascular diseases, sex hormone disorders, metabolic diseases, LUTSs, obstructive sleep apnea syndrome, and psychosocial diseases, among others, which are all important factors of the cause or aggravation of ED. At the same time, these comorbidities increase the difficulty of ED treatment and are important factors for the lack of a response to PDE5is. In the course of ED treatment, the treatment of comorbidities will improve the efficacy of the ED treatment. Nevertheless, the side effects of some drugs used to treat comorbidities can impair erectile function. Interestingly, some drugs combined with PDE5is have synergistic effects in treating ED. Overall, understanding the effects of drugs for comorbidities on ED will help in the development of therapeutic strategies for PDE5i nonresponders.

##### 3.4.2.1 Associated medication modifications

It is worth noting that while treating primary diseases or comorbidities, the side effects of some drugs can impair male erectile function. Antidepressants, such as selective serotonin-reuptake inhibitors and tricyclic antidepressants, antipsychotic drugs, such as phenothiazines and butanones, antihypertensive drugs, such as thiazides and β-blockers, and lipid-lowering drugs, such as statins and antiandrogens, can lead to male ED [106-107]. In the course of ED treatment, the use of these drugs will aggravate the difficulty treating ED. The drugs taken by patients with ED should be considered, and associated medications should be modified to reduce the loss of therapeutic effects on ED.

##### 3.4.2.2 PDE5is combined with other non-PDE5i drugs

For ED patients with other disorders, the therapeutic regimen focuses on the control or cure of the primary diseases, although the application of PDE5is at suitable doses for an adequate course of treatment, regardless of these diseases, is a definite factor related to the status of ED. Therefore, combining PDE5is with other drugs for comorbidities is an important strategy for improving ED or salvaging erectile function in PDE5i nonresponders.

**Figure 1.**
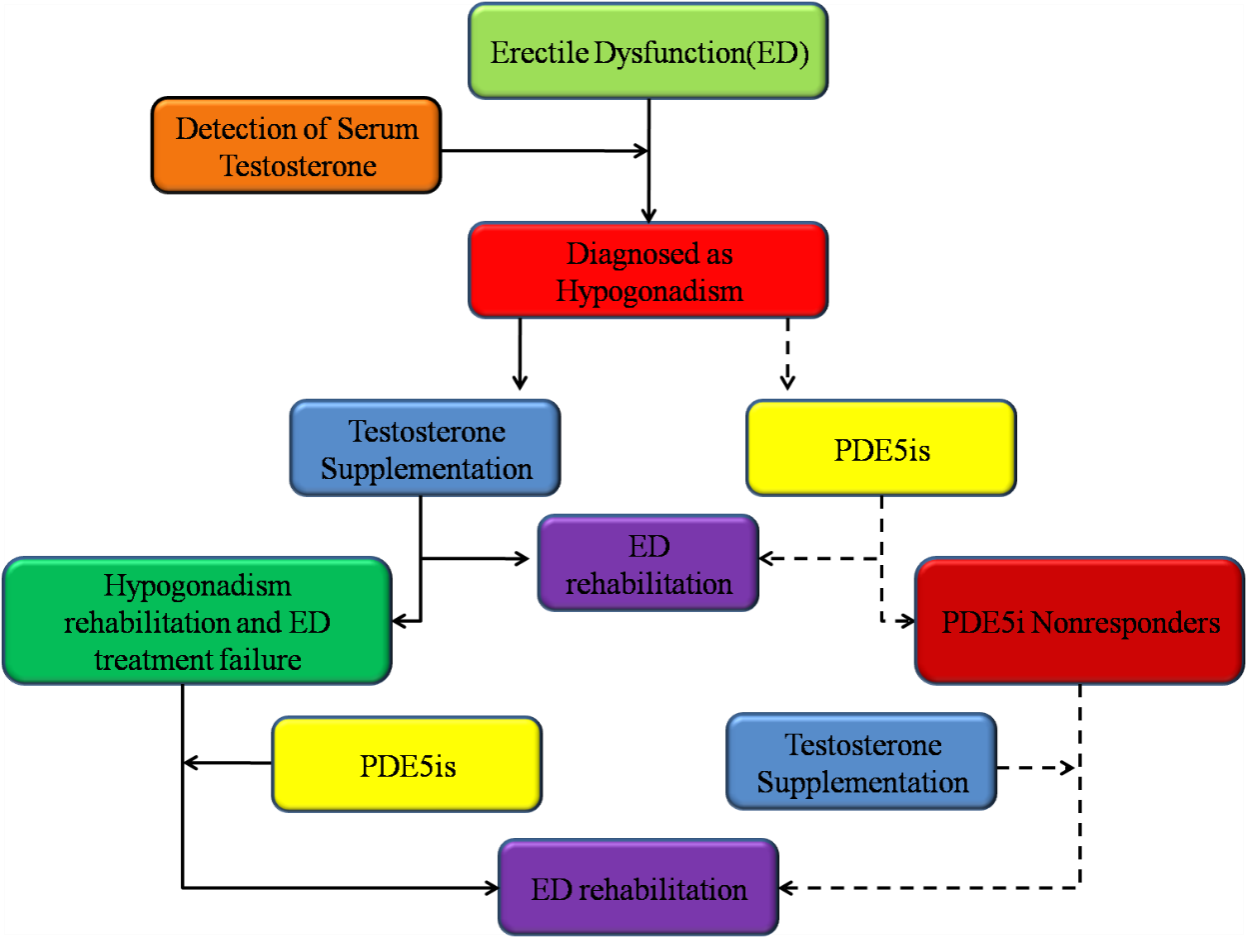
Management of patients with ED and hypogonadism. In patients with ED, more attention should be paid to testosterone supplementation after hypogonadism is confirmed by the detection of testosterone. In patients with ED and hypogonadism, some will recover from ED after treatment with PDE5is, but others will show nonresponsiveness to PDE5is; in these cases, ED can be treated by adding testosterone to the PDE5i treatment. The reason for this lack of a response is that PDE5 is under the control of testosterone, and a normal testosterone level is the basis for the full effect of PDE5is. Therefore, in ED patients with hypogonadism, we recommend giving priority to testosterone supplementation to treat a portion of them and using a combination of testosterone supplementation and PDE5is for the remaining patients.

*In vitro* studies have found that persistent low testosterone levels can lead to a decrease in the activity and expression of NO synthase (NOS), thereby damaging penile tissues [108]; normal testosterone levels result in PDE5i efficacy because PDE5 is under the control of testosterone [109]. Serum testosterone levels less than 300 ng/dl can hamper the response to PDE5is [110]. In patients with ED and hypogonadism, which leads to low testosterone levels, testosterone alone can enable 34.4% of patients to achieve satisfactory erection, with the combined use of testosterone and sildenafil allowing another 37.5% patients to achieve satisfactory erection. These results suggest that the combination of testosterone and sildenafil has synergism in ED [111]. Additionally, testosterone combined with 10 mg of tadalafil once a day or 20 mg twice a week can treat ED in patients with low testosterone levels (T ≤3 or 3.4 ng/ mL) who do not respond to tadalafil [110, 112-113]. Therefore, during treatment with PDE5is for ED along with hypogonadism, the addition of testosterone can rescue PDE5i nonresponsiveness. For routine treatment, testosterone alone can be used first in ED patients with hypogonadism. If this is not sufficient to restore erection, a short-acting PDE5i (sildenafil or vardenafil) can be added and, if necessary, combined with a long-acting PDE5i (tadalafil) [109] (Fig. 1). Middle-aged and elderly men commonly always suffer from BPH, which increases the risk of ED [114], and α-adrenergic antagonists are commonly used for treating BPH. Evidence-based studies have found that in patients with BPH and ED, the combination of a PDE5i and an α-adrenergic antagonist resulted in higher IIEF scores compared with the use of a PDE5i alone [115]; in particular, the long-term use of long-acting tadalafil was found to be remarkably beneficial for ED along with BPH/LUTSs [109]. Statins are important lipid-lowering drugs, and their side effects can cause male dysfunction. Interestingly, by activating endogenous NOS, atorvastatin significantly elevated the IIEF-5 score and improved erectile function in patients with ED who did not respond to sildenafil [116]. Two clinical studies have found that adding atorvastatin to sildenafil for ED significantly improved the response to sildenafil and enhanced erectile function in patients with hypercholesterolemia and no response to sildenafil [117-118]. In conclusion, combining non-PDE5i drugs with PDE5is to improve the therapeutic effect on ED in PDE5i nonresponders is an important strategy to consider. It is also worth noting that in studies of sildenafil nonresponders, some drugs, such as intraurethral alprostadil, cabergoline, and intracavernous PGE1 injections, were found to restore erectile function in patients who did not respond to sildenafil treatment [119-121]. Although there have been no studies to date on these drugs in combination with PDE5is for ED in PDE5i nonresponders, these drugs may also represent important future directions for ED treatment.

### 3.5 Lifestyle adjustments: a neglected issue

In the process of using PDE5is to treat ED, lifestyle adjustments, such as those involving smoking, drinking, low levels of physical activity, and obesity, are important factors affecting erectile function and the efficacy of these drugs. Smoking increases the risk for ED development, regardless of whether it is active or passive smoking [122]. Compared to a nonsmoking group, a (current and past) smoking group showed an odds ratio (OR) for ED of 1.51 (95% CI: 1.34-1.71), whereas the OR for ED in the group with a past smoking history was 1.29 (95% CI: 1.07-1.47) [123]. Indeed, the cessation of smoking is reported to enhance sexual health indexes in male smokers with a relatively long smoking history, and this effect was independent of the current ED status. Long-term and excessive drinking are also risk factors for sexual dysfunction [124]. For instance, it has been demonstrated that heavy drinking and nondrinking are associated with the ED risk and that moderate drinking actually protects males from developing ED and diabetes [125-126]. Alcohol consumption can also seriously affect the efficacy of sildenafil in the treatment of ED, which is related to the pharmacological characteristics of this drug. Overweight, obesity and metabolic syndrome are all associated with an increased risk of ED [127]. In one study, the risk of ED was increased to 40% in males with obesity [125], and sexual activity and behaviors improved in ED patients who participated in a weight-loss program [128]. Some studies have also reported that adherence to the Mediterranean diet could help patients with obesity and metabolic syndrome alleviate and resolve symptoms of ED in comparison with the controls [129]. Moreover, a high-fat meal slows the absorption of most PDE5is, except tadalafil and the orally disintegrating formulation of vardenafil, delaying the maximum blood concentration for up to approximately 1 h. Thus, some ED patients who have sex according to the drug instructions may inadvertently unconsciously and indirectly reduce the therapeutic effect due to an insufficient blood drug concentration [12]. It has been found that a low exercise level is an independent factor that correlates positively with the risk of ED [130-131]. A study on the mechanism involved found that with increasing exercise, markers of vascular endothelial dysfunction, such as serum endothelial progenitor cells and endothelial microparticles, decreased significantly, suggesting that exercise can improve erectile function by protecting vascular endothelial cells [67]. In short, ED patients treated with PDE5is should be advised to adopt a healthy lifestyle as part of their regimen. Although there have been no studies on the direct relationship between lifestyle adjustments and PDE5i nonresponsiveness, lifestyle adjustments may be a nonnegligible strategy in these patients.

### 3.6 Patient management

Counseling based on the needs of patients and their partners is an important part of ED management. The approach requires good communication between patients and doctors, which helps to resolve patients' concerns and facilitate implementation of the clinicians' recommendations [12]. Additionally, there are many factors affecting nonresponsiveness to PDE5is during ED treatment. For example, due to the pharmacokinetic characteristics of PDE5is, most should be taken 45-60 minutes before sexual intercourse, and premature sexual activity after taking a PDE5i will lead to a poor therapeutic effect due to an insufficient blood concentration [12]. A high-fat diet can also delay the maximum observed concentration of most PDE5is, except tadalafil and the orally disintegrating formulation of vardenafil, and thus affect the treatment outcome [132]. Furthermore, some patients also use herbal remedies or dietary supplements during ED treatment. Although some are effective, these medicines contain many unknown ingredients [12], and they may increase the difficulty of using PDE5is in nonresponders. Therefore, periodic follow-up visits with patients can help to reveal the use of such drugs, the psychological state and the medical status to determine whether the treatment is adequate, whether the medications are correct, whether poor lifestyle factors have been addressed, and whether there are other adverse factors affecting ED treatment to guide the correct management of ED [12, 22, 29].

## 4. Management of strategies for treating ED in PDE5i nonresponders

**Figure 2.**
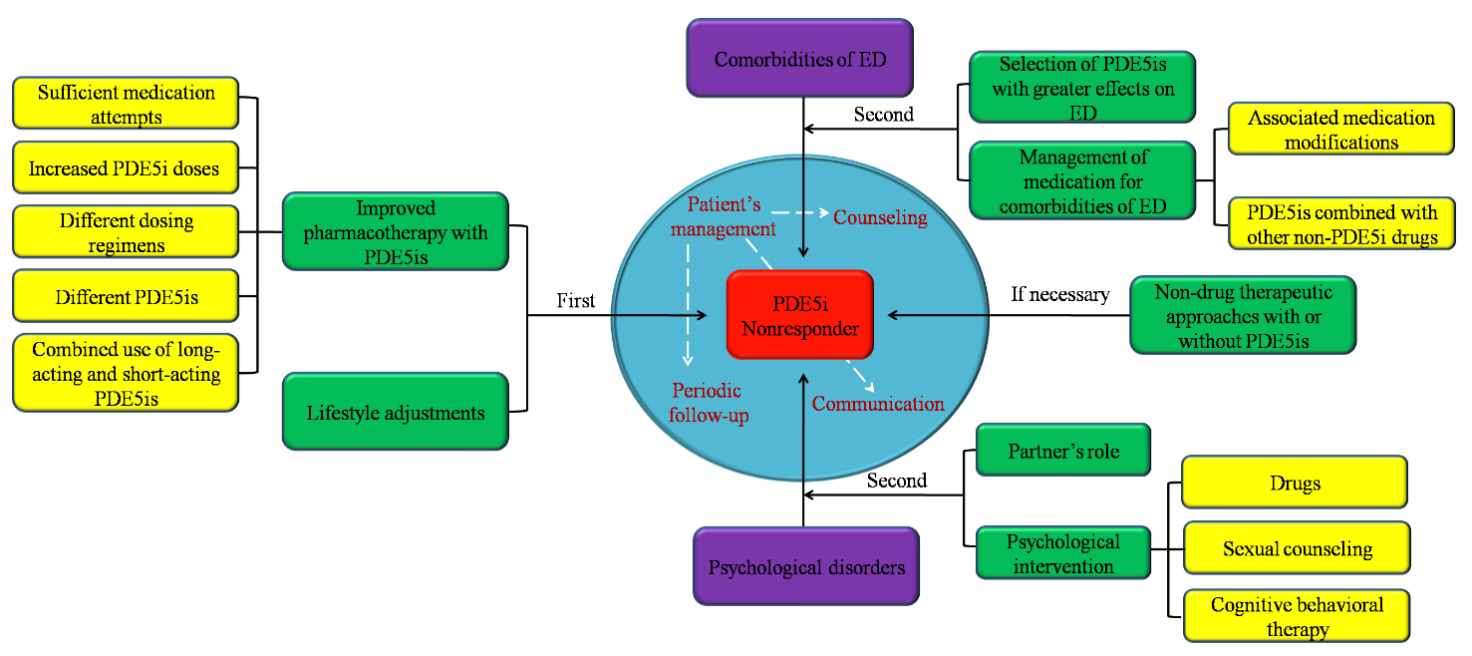
Management of strategies to treat ED in PDE5i nonresponders. In the management of PDE5i nonresponders, the first-line strategies consist of lifestyle adjustments and improved pharmacotherapy with PDE5is, including sufficient medication attempts, increased PDE5i doses, different dosing regimens, different PDE5is and the combined use of long-acting and short-acting PDE5is. If the patient has an obvious mental disorder, we should focus on the patient’s psychology and give corresponding treatment, such as attaching importance to the partner’s role and providing psychological intervention, including drugs, sexual counseling and cognitive behavioral therapy. In addition, strategies of improved pharmacotherapy with PDE5is and lifestyle adjustments should be added. If ED patients have comorbidities, comorbidity-related strategies, such as the selection of PDE5is with greater effects on ED and the management of medications for comorbidities of ED, including associated medication modifications, and combining PDE5is with other non-PDE5i drugs, should be fully considered on the basis of the strategies of improved pharmacotherapy with PDE5is and lifestyle adjustments. If necessary, non-drug therapeutic approaches with or without PDE5is can be selected according to the actual treatment profile of each PDE5i nonresponder. It is worth noting that in process of treating every PDE5i nonresponder, patient management should be of great concern. Periodic follow-up visits should be carried out to find any deficiencies in the ED treatment process. Good communication should also be established through patient counseling to resolve patients' concerns and ensure the smooth implementation of treatment.

In the management of ED treatment, lifestyle factors, such as smoking, excessive drinking, high-fat diets, and low exercise levels are closely related to the treatment effect; thus, addressing the above unhealthy lifestyle factors is necessary in the treatment of PDE5i nonresponders. Accordingly, lifestyle adjustments combined with improved pharmacotherapy with PDE5is is the first choice. At the same time, for patients with definite mental issues, therapeutic strategies to improve the psychological status should be added. For ED patients with comorbidities, the treatment of these comorbidities is an important prerequisite to ensure and improve the therapeutic effects on ED. Therefore, on the basis of correcting unhealthy lifestyle factors and improving PDE5i treatment, cooperation with comorbidity-related treatment strategies should be instilled. Of course, disease characteristics vary among ED patients, and individualized treatment should be emphasized. In particular, for the treatment of ED patients who are nonresponsive to PDE5is, the different treatment strategies mentioned above should be jointly adopted according to the characteristics of each patient (Fig. 2). It is noteworthy that there is a difference between the application of treatment approaches in young adults and elderly men. In young adults, psychological factors have a great impact on ED, and attention should be directed toward strengthening the treatment of these factors to quickly improve erectile function. In older men, the presence of comorbidities due to aging may be the main reason for the difficulty in treating ED, and attention should be directed toward overall health and the management of chronic diseases in these ED patients.

## 5. Conclusions

ED is a common sexual abnormality in adult men that measurably affects their psychological and physical QOL as well as that of their partners. The discovery and application of PDE5is has been revolutionary for the treatment of ED. With increasing clinical trials on PDE5is and ED, there are now many strategies for improving erectile function, as reviewed above. Regardless, ED is a very complex condition involving both psychological and physical factors. The treatment program for ED will vary among individuals, especially in patients with a poor response to PDE5is. Therefore, combinations of various strategies to enhance the response to PDE5is should be applied to improve the therapeutic efficacy according to the clinical characteristics of each patient. Additionally, PDE5i nonresponders are a unique group of ED patients, and the causes of the lack of a response to PDE5is remain to be clarified. Although some studies have suggested improved strategies for PDE5i nonresponders, these studies are limited, and more related basic and clinical research is needed to help PDE5i nonresponders achieve satisfactory sexual experiences.

## References

[1] Allen MS, Walter EE (2019). Erectile Dysfunction: An Umbrella Review of Meta-Analyses of Risk-Factors, Treatment, and Prevalence Outcomes. J Sex Med, pii: S1743-6095(19)30354-6. 10.1016/j.jsxm.2019.01.314 30833150

[2] Lin H, Wang T, Ruan Y, Liu K, Li H, Wang S, et al(2018). Rapamycin Supplementation May Ameliorate Erectile Function in Rats With Streptozotocin-Induced Type 1 Diabetes by Inducing Autophagy and Inhibiting Apoptosis, Endothelial Dysfunction, and Corporal Fibrosis. J Sex Med, 15(9):1246-1259. 30224017 10.1016/j.jsxm.2018.07.013

[3] Li H, Gao T, Wang R (2016). The role of the sexual partner in managing erectile dysfunction. Nat Rev Urol, 13:168-77. 26832165 10.1038/nrurol.2015.315

[4] Geerkens MJM, Al-Itejawi HHM, Nieuwenhuijzen JA, Meuleman EJM, Lissenberg-Witte BI, van Moorselaar RJA, et al (2019). Sexual Dysfunction and Bother Due to Erectile Dysfunction in the Healthy Elderly Male Population: Prevalence from a Systematic Review. Eur Urol Focus. pii: S2405-4569(19)30079-3. 10.1016/j.euf.2019.03.004 30878347

[5] Moncada I, Martinez-Salamanca J, Ruiz-Castañe E, Romero J (2018). Combination therapy for erectile dysfunction involving a PDE5 inhibitor and alprostadil. Int J Impot Res, 30(5):203-208. 30050072 10.1038/s41443-018-0046-2

[6] Hawksworth DJ, Burnett AL (2015). Pharmacotherapeutic management of erectile dysfunction. Clin Pharmacol Ther, 98(6):602-10. 26352006 10.1002/cpt.261

[7] McMahon CN, Smith CJ, Shabsigh R (2006). Treating erectile dysfunction when PDE5 inhibitors fail. BMJ, 332: 589-92 16528082 10.1136/bmj.332.7541.589 PMC1397768

[8] Moon KH, Park SY, Kim YW (2019). Obesity and Erectile Dysfunction: From Bench to Clinical Implication. World J Mens Health, 37(2):138-147. 30079640 10.5534/wjmh.180026 PMC6479091

[9] Albersen M, Mwamukonda KB, Shindel AW, Lue TF (2011). Evaluation and treatment of erectile dysfunction. Med Clin North Am, 95(1):201-12. 21095423 10.1016/j.mcna.2010.08.016

[10] Milenkovic U, Campbell J, Roussel E, Albersen M (2018). An update on emerging drugs for the treatment of erectile dysfunction. Expert Opin Emerg Drugs, 23(4):319-330. 30507329 10.1080/14728214.2018.1552938

[11] Echeverri Tirado LC, Ferrer JE, Herrera AM (2016). Aging and Erectile Dysfunction. Sex Med Rev, 4(1):63-73. 27872006 10.1016/j.sxmr.2015.10.011

[12] Mulhall JP, Giraldi A, Hackett G, Hellstrom WJG, Jannini EA, Rubio-Aurioles E, et al (2018). The 2018 Revision to the Process of Care Model for Management of Erectile Dysfunction. J Sex Med, 15(10):1434-1445. 30057278 10.1016/j.jsxm.2018.05.021

[13] McCabe MP, Sharlip ID, Lewis R, Atalla E, Balon R, Fisher AD, et al (2016). Incidence and prevalence of sexual dysfunction in women and men: a consensus statement from the Fourth International Consultation on Sexual Medicine 2015. J Sex Med, 13:144-152. 26953829 10.1016/j.jsxm.2015.12.034

[14] Ayta IA, McKinlay JB, Krane RJ (1999). The likely worldwide increase in erectile dysfunction between 1995 and 2025 and some possible policy consequences. BJU Int, 84:50. 10444124 10.1046/j.1464-410x.1999.00142.x

[15] Goldstein I, Chambers R, Tang W, Stecher V, Hassan T (2018). Real-world observational results from a database of 48 million men in the United States: Relationship of cardiovascular disease, diabetes mellitus and depression with age and erectile dysfunction. Int J Clin Pract, 72(4):e13078. 29569323 10.1111/ijcp.13078

[16] Schulster ML, Liang SE, Najari BB (2017). Metabolic syndrome and sexual dysfunction. Curr Opin Urol, 27(5):435-440. 28650864 10.1097/MOU.0000000000000426

[17] Bilgutay AN, Pastuszak AW (2015). PEYRONIE'S DISEASE: A REVIEW OF ETIOLOGY, DIAGNOSIS, AND MANAGEMENT. Curr Sex Health Rep, 7(2):117-131. 26279643 10.1007/s11930-015-0045-y PMC4535719

[18] Pavone C, D'Amato F, Dispensa N, Torretta F, Magno C (2015). Smoking, diabetes, blood hypertension: possible etiologic role for Peyronie’s disease? Analysis in 279 patients with a control group in Sicily. Arch Ital Urol Androl, 87(1):20-4. 25847891 10.4081/aiua.2015.1.20

[19] Herrero A, Marcos MGalindo P (2018). Clinical and biochemical correlates of male hypogonadism in type 2 diabetes. Andrology, 6(1):58-63. 29145714 10.1111/andr.12433

[20] Zhang J, Li X, Cai Z, Li H, Yang B (2019). Association between testosterone with type 2 diabetes in adult males, a meta-analysis and trial sequential analysis. Aging Male, 16:1-12. 10.1080/13685538.2018.1557139 30651030

[21] Anderson R, Moses R, Lenherr S, Hotaling JM, Myers J (2018). Spinal cord injury and male infertility-a review of current literature, knowledge gaps, and future research. Transl Androl Urol, 7(Suppl 3):S373-S382. 30159244 10.21037/tau.2018.04.12 PMC6087847

[22] Atiemo HO, Szostak MJ, Sklar GN (2003). Salvage of sildenafil failures referred from primary care physicians. J Urol, 170(6 Pt 1):2356-8. 14634415 10.1097/01.ju.0000096221.67967.ae

[23] Korakas E, Dimitriadis G, Raptis A, Lambadiari V (2018). Dietary Composition and Cardiovascular Risk: A Mediator or a Bystander? Nutrients, 10(12). pii: E1912. 10.3390/nu10121912 PMC6316552 30518065

[24] Poudel A, Zhou JY, Story D, Li L (2018). Diabetes and Associated Cardiovascular Complications in American Indians/Alaskan Natives: A Review of Risks and Prevention Strategies. J Diabetes Res, 2018:2742565. 30302343 10.1155/2018/2742565 PMC6158951

[25] Szafran-Dobrowolska J, Renke M, Jeżewska M (2019). Is it worth to continue to analyse the factors of cardiovascular risk among the sailors? Review of literature. Int Marit Health, 70(1):17-21. 30931513 10.5603/IMH.2019.0003

[26] Chiang PK, Yang FY (2019). A potential treatment of low intensity pulsed ultrasound on cavernous nerve injury for erectile dysfunction. Med Hypotheses, 122:19-21. 30593410 10.1016/j.mehy.2018.10.014

[27] Barazani Y, Stahl PJ, Nagler HM, Stember DS (2015). Is there a rationale for penile rehabilitation following radical prostatectomy? Am J Mens Health, 9(1):35-43. 24692247 10.1177/1557988314528237

[28] Fenstermaker M, Dupree JM, Hadj-Moussa M, Ohl DA (2018). Management of Erectile Dysfunction and Infertility in the Male Spinal Cord Injury Patient. Curr Urol Rep, 19(7):47. 29774436 10.1007/s11934-018-0806-z

[29] Jiann BP, Yu CC, Su CC, Huang JK (2004). Rechallenge prior sildenafil nonresponders. Int J Impot Res, 16(1):64-8. 14963473 10.1038/sj.ijir.3901143

[30] Jackson SE, Firth J, Veronese N, Stubbs B, Koyanagi A, Yang L, et al (2019). Decline in sexuality and wellbeing in older adults: A population-based study. J Affect Disord, 245:912-917. 30699876 10.1016/j.jad.2018.11.091

[31] Khan S, Amjad A, Rowland D (2019). Potential for Long-Term Benefit of Cognitive Behavioral Therapy as an Adjunct Treatment for Men with Erectile Dysfunction. J Sex Med, 16(2):300-306. 30770073 10.1016/j.jsxm.2018.12.014

[32] Shallcross AJ, Willroth EC, Fisher A, Dimidjian S, Gross JJ, Visvanathan PD, et al (2018). Relapse/Recurrence Prevention in Major Depressive Disorder: 26-Month Follow-Up of Mindfulness-Based Cognitive Therapy Versus an Active Control. Behav Ther, 49(5):836-849. 30146148 10.1016/j.beth.2018.02.001 PMC6112178

[33] McCullough AR, Barada JH, Fawzy A, Guay AT, Hatzichristou D (2002). Achieving treatment optimization with sildenafil citrate (Viagra) in patients with erectile dysfunction. Urology, 60(2 Suppl 2): 28-38. 10.1016/s0090-4295(02)01688-6 12414331

[34] Hellstrom WJ, Elhilali M, Homering M, Taylor T, Gittleman M (2005). Vardenafil in patients with erectile dysfunction: achieving treatment optimization. J Androl, 26(5):604-9. 16088037 10.2164/jandrol.05026

[35] Sontag A, Rosen RC, Litman HJ, Ni X, Araujo AB (2013). The role of initial success rates and other factors in determining reliability of outcomes of phosphodiesterase inhibitor therapy for erectile dysfunction: a pooled analysis of 17 placebo-controlled trials of tadalafil for use as needed. J Sex Med, 10(2):541-50. 22905853 10.1111/j.1743-6109.2012.02901.x

[36] Steidle CP, McCullough AR, Kaminetsky JC, Crowley AR, Siegel RL, Deriesthal H, et al (2007). Early sildenafil dose optimization and personalized instruction improves the frequency, flexibility, and success of sexual intercourse in men with erectile dysfunction. Int J Impot Res, 19(2):154-60. 16858367 10.1038/sj.ijir.3901498

[37] Montorsi F, McCullough A (2005). Efficacy of sildenafil citrate in men with erectile dysfunction following radical prostatectomy: a systematic review of clinical data. J Sex Med, 2(5):658-67. 16422824 10.1111/j.1743-6109.2005.00117.x

[38] McMahon CG (2002). High dose sildenafil citrate as a salvage therapy for severe erectile dysfunction. Int J Impot Res, 14(6):533-8. 12494291 10.1038/sj.ijir.3900936

[39] Shin YS, Lee SW, Park K, Chung WS, Kim SW, Hyun JS, et al (2015). Effect of levitra on sustenance of erection (EROS): an open-label, prospective, multicenter, single-arm study to investigate erection duration measured by stopwatch with flexible dose vardenafil administered for 8 weeks in subjects with erectile dysfunction. Int J Impot Res, 27(3):95-102. 25471318 10.1038/ijir.2014.39

[40] McMahon C, Lording D, Stuckey B, Tan V, Gillman M, White W, et al (2006). Vardenafil improved erectile function in a "real-life" broad population study of men with moderate to severe erectile dysfunction in Australia and New Zealand. J Sex Med, 3(5):892-900. 16942533 10.1111/j.1743-6109.2006.00296.x

[41] Porst H, Gacci M, Büttner H, Henneges C, Boess F (2014). Tadalafil once daily in men with erectile dysfunction: an integrated analysis of data obtained from 1913 patients from six randomized, double-blind, placebo-controlled, clinical studies. Eur Urol, 65(2):455-64. 24119319 10.1016/j.eururo.2013.09.037

[42] Lee JG, Kim BD, Han CH, Lee KK, Yum KS (2018). Evaluation of the effectiveness and safety of a daily dose of 5?mg of tadalafil, over an 8-week period, for improving quality of life among Korean men with andropause symptoms, including erectile dysfunction: A pilot study. Medicine (Baltimore), 97(51):e13827. 30572547 10.1097/MD.0000000000013827 PMC6320193

[43] Jiang H, Zhao LM, Lin HC, Yan S, Liu JH, Zhu ZH, et al (2018). Evaluation of the long-term safety and effectiveness of tadalafil once daily in Chinese men with erectile dysfunction: interim results of a multicenter, randomized, open-label trial. Asian J Androl, 20(6):587-592. 30004039 10.4103/aja.aja_47_18 PMC6219303

[44] Carrier S, Brock GB, Pommerville PJ, Shin J, Anglin G, Whitaker S, Beasley CM Jr (2005). Efficacy and safety of oral tadalafil in the treatment of men in Canada with erectile dysfunction: a randomized, double-blind, parallel, placebo-controlled clinical trial. J Sex Med, 2(5):685-98. 16422827 10.1111/j.1743-6109.2005.00097.x

[45] Romero Otero J, García Gómez B, Medina Polo J, Jiménez Alcaide E, García Cruz E, Sallent Font A, et al (2014) Evaluation of current errors within the administration of phosphodiesterase-5 inhibitors after more than 10 years of use. Urology, 83:1334-1338. 24745797 10.1016/j.urology.2014.02.016

[46] McMahon C (2004). Efficacy and safety of daily tadalafil in men with erectile dysfunction previously unresponsive to on-demand tadalafil. J Sex Med, 1(3):292-300. 16422959 10.1111/j.1743-6109.04042.x

[47] Javaroni V, Queiroz Miguez M, Burla A, Oigman W, Neves MF (2012). Response to on-demand vardenafil was improved by its daily usage in hypertensive men. Urology, 80(4):858-64. 22921698 10.1016/j.urology.2012.06.042

[48] Brisson TE, Broderick GA, Thiel DD, Heckman MG, Pinkstaff DM (2006). Vardenafil rescue rates of sildenafil nonresponders: objective assessment of 327 patients with erectile dysfunction. Urology, 68(2):397-401. 16904460 10.1016/j.urology.2006.03.005

[49] Hatzichristou DG, Aliotta P, Auerbach S, Barkin J, Lording D, Murdock M, et al (2005). Erectile response to vardenafil in men with a history of nonresponse to sildenafil: a time-from-dosing descriptive analysis. Clin Ther, 27(9):1452-61. 16291418 10.1016/j.clinthera.2005.09.014

[50] Rubio-Aurioles E, Glina S, Abdo CH, Hernandez-Serrano R, Rampazzo C, Sotomayor M, et al (2009) Timing of dose relative to sexual intercourse attempt in previous sildenafil citrate users treated with tadalafil: a geographical comparison from a single arm, open-label study. J Sex Med, 6(10):2836-50. 19674256 10.1111/j.1743-6109.2009.01413.x

[51] Li HJ, Bai WJ, Dai YT, Xu WP, Wang CN, Li HZ (2016). An analysis of treatment preferences and sexual quality of life outcomes in female partners of Chinese men with erectile dysfunction. Asian J Androl, 18(5):773-9. 26459780 10.4103/1008-682X.159719 PMC5000803

[52] Ahn TY, Lee SW, Kim SW, Yang DY, Park NC, Min KS, et al (2007). Treatment preferences in men with erectile dysfunction: an open label study in Korean men switching from sildenafil citrate to tadalafil. Asian J Androl, 9(6):760-70. 17968461 10.1111/j.1745-7262.2007.00319.x

[53] Ozgur BC, Gonenc F, Yazicioglu AH (2009). Sildenafil or vardenafil nonresponders' erectile response to tadalafil. Urol J, 6(4):267-71. 20027555

[54] Corona G, Rastrelli G, Burri A, Jannini EA, Maggi M (2016). The safety and efficacy of Avanafil, a new 2(nd) generation PDE5i: comprehensive review and meta-analysis. Expert Opin Drug Saf, 15(2):237-47. 26646748 10.1517/14740338.2016.1130126

[55] Caretta N, Palego P, Ferlin A, Garolla A, Bettella A, Selice R, et al (2005). Resumption of spontaneous erections in selected patients affected by erectile dysfunction and various degrees of carotid wall alteration: role of tadalafil. Eur Urol, 48(2):326-31. 16005378 10.1016/j.eururo.2005.01.013

[56] Cui H, Liu B, Song Z, Fang J, Deng Y, Zhang S, et al (2015). Efficacy and safety of long-term tadalafil 5 mg once daily combined with sildenafil 50 mg as needed at the early stage of treatment for patients with erectile dysfunction. Andrologia, 47(1):20-4. 24387078 10.1111/and.12216

[57] Sun L, Peng FL, Yu ZL, Liu CL, Chen J (2014). Combined sildenafil with vacuum erection device therapy in the management of diabetic men with erectile dysfunction after failure of first-line sildenafil monotherapy. Int J Urol, 21(12):1263-7. 25039272 10.1111/iju.12564

[58] Chen J, Sofer M, Kaver I, Matzkin H, Greenstein A (2004). Concomitant use of sildenafil and a vacuum entrapment device for the treatment of erectile dysfunction. J Urol, 171(1):292-5. 14665897 10.1097/01.ju.0000098460.02560.fe

[59] Canguven O, Bailen J, Fredriksson W, Bock D, Burnett AL (2009). Combination of vacuum erection device and PDE5 inhibitors as salvage therapy in PDE5 inhibitor nonresponders with erectile dysfunction. J Sex Med, 6(9):2561-7. 19627462 10.1111/j.1743-6109.2009.01364.x

[60] Srini VS, Reddy RK, Shultz T, Denes B (2015). Low intensity extracorporeal shockwave therapy for erectile dysfunction: a study in an Indian population. Can J Urol, 22(1):7614-22. 25694008

[61] Brunckhorst O, Wells L, Teeling F, Muir G, Muneer A, Ahmed K (2019). A systematic review of the long-term efficacy of low-intensity shockwave therapy for vasculogenic erectile dysfunction. Int Urol Nephrol, 51(5):773-781. 30903393 10.1007/s11255-019-02127-z PMC6499893

[62] Wang CJ, Lu YM, Li CC, Wu WJ, Chien TM (2019). Low-intensity shock wave therapy ameliorates erectile dysfunction in men with pelvic fractures associated with urethral injury. Int J Impot Res, 31(3):218-222. 30420772 10.1038/s41443-018-0094-7

[63] Bechara A, Casabé A, De Bonis W, Ciciclia PG (2016). Twelve-Month Efficacy and Safety of Low-Intensity Shockwave Therapy for Erectile Dysfunction in Patients Who Do Not Respond to Phosphodiesterase Type 5 Inhibitors. Sex Med, 4(4):e225-e232. 27444215 10.1016/j.esxm.2016.06.001 PMC5121537

[64] Tsai CC, Wang CJ, Lee YC, Kuo YT, Lin HH, Li CC, et al (2017). Low-intensity extracorporeal shockwave therapy can improve erectile function in patients who failed to respond to phosphodiesterase type 5 inhibitors. Am J Mens Health, 11(6):1781-90. 28884638 10.1177/1557988317721643 PMC5675264

[65] Chen S, Peng D, Xu X, Gao J, Dai F, Zuo C, et al (2017). Assessment of erectile dysfunction and associated psychological distress in Chinese men with type 2 diabetes mellitus. Int J Impot Res, 29(5):210-214. 28659632 10.1038/ijir.2017.25

[66] Cheng CM, Lin YC, Chang KC (2017). Psychological Distress is Correlated with Erectile Dysfunction Among Patients Receiving Methadone Maintenance in Taiwan. J Dual Diagn, 13(4):312-316. 29120281 10.1080/15504263.2017.1363449

[67] Hehemann MC, Kashanian JA (2016). Can lifestyle modification affect men’s erectile function? Transl Androl Urol, 5(2):187-94. 27141445 10.21037/tau.2016.02.05 PMC4837314

[68] Taneja R (2007). A rational combination pharmacotherapy in men with erectile dysfunction who initially failed to oral sildenafil citrate alone: a pilot study. J Sex Med, 4(4 Pt 2):1136-41. 17484772 10.1111/j.1743-6109.2007.00507.x

[69] Chambers SK, Occhipinti S, Schover L, Nielsen L, Zajdlewicz L, Clutton S, et al (2015). A randomised controlled trial of a couples-based sexuality intervention for men with localised prostate cancer and their female partners. Psychooncology, 24(7):748-56. 25483780 10.1002/pon.3726

[70] Pisano F, Falcone M, Abbona A, Oderda M, Soria F, Peraldo F, et al (2015). The importance of psychosexual counselling in the re-establishment of organic and erotic functions after penile prosthesis implantation. Int J Impot Res, 27(5):197-200. 26268774 10.1038/ijir.2015.17

[71] Nowroozi MR, Amini E, Ayati M, Jamshidian H, Radkhah K, Amini S (2015). Applying extender devices in patients with penile dysmorphophobia: assessment of tolerability, efficacy, and impact on erectile function. J Sex Med, 12(5):1242-7. 25809129 10.1111/jsm.12870

[72] Chertin B, Natsheh A, Ben-Zion I, Prat D, Kocherov S, Farkas A, et al (2013). Objective and subjective sexual outcomes in adult patients after hypospadias repair performed in childhood. J Urol, 190(4 Suppl):1556-60. 23306088 10.1016/j.juro.2012.12.104

[73] Chambers SK, Occhipinti S, Stiller A, Zajdlewicz L, Nielsen L, Wittman D, et al (2019). Five-year outcomes from a randomised controlled trial of a couples-based intervention for men with localised prostate cancer. Psychooncology, 28(4):775-783. 30716188 10.1002/pon.5019

[74] White ID, Wilson J, Aslet P, Baxter AB, Birtle A, Challacombe B, et al (2015). Development of UK guidance on the management of erectile dysfunction resulting from radical radiotherapy and androgen deprivation therapy for prostate cancer. Int J Clin Pract, 69(1):106-23. 25283500 10.1111/ijcp.12512 PMC4309408

[75] Banner LL, Anderson RU (2007). Integrated sildenafil and cognitive-behavior sex therapy for psychogenic erectile dysfunction: a pilot study. J Sex Med, 4(4 Pt 2):1117-25. 17627724 10.1111/j.1743-6109.2007.00535.x

[76] Giuri S, Caselli G, Manfredi C, Rebecchi D, Granata A, Ruggiero GM, et al (2017). Cognitive Attentional Syndrome and Metacognitive Beliefs in Male Sexual Dysfunction: An Exploratory Study. Am J Mens Health, 11(3):592-599. 27283433 10.1177/1557988316652936 PMC5675226

[77] Wittmann D, Carolan M, Given B, Skolarus TA, An L, Palapattu G, et al (2014). Exploring the role of the partner in couples' sexual recovery after surgery for prostate cancer. Support Care Cancer, 22:2509-15. 24728619 10.1007/s00520-014-2244-x

[78] Bai WJ, Li HJ, Dai YT, He XY, Huang YR, Liu JH, et al (2015). An open-label, multicenter, randomized, crossover study comparing sildenafil citrate and tadalafil for treating erectile dysfunction in Chinese men naive to phosphodiesterase 5 inhibitor therapy. Asian J Androl, 17:61-7. 25370206 10.4103/1008-682X.143244 PMC4291880

[79] Chen L, Shi GR, Huang DD, Li Y, Ma CC, Shi M, et al (2019). Male sexual dysfunction: A review of literature on its pathological mechanisms, potential risk factors, and herbal drug intervention. Biomed Pharmacother, 112:108585. 30798136 10.1016/j.biopha.2019.01.046

[80] Jannini EA, Lombardo F, Lenzi A (2005). Correlation between ejaculatory and erectile dysfunction. Int J Androl, 28(2 suppl):40-5. 16236063 10.1111/j.1365-2605.2005.00593.x

[81] el-Sakka AI (2008). Severity of erectile dysfunction at presentation: effect of premature ejaculation and low desire. Urology, 71: 94-98. 18242373 10.1016/j.urology.2007.09.006

[82] Jannini EA, McMahon C, Chen J, Aversa A, Perelman M (2011). The controversial role of phosphodiesterase type 5 inhibitors in the treatment of premature ejaculation. J Sex Med, 8(8):2135-43. 21791006 10.1111/j.1743-6109.2011.02401.x

[83] Jannini EA, Lenzi A, Isidori A, Fabbri A (2006). Subclinical erectile dysfunction: Proposal for a novel taxonomic category in sexual medicine. J Sex Med, 3(5):787-794. 16942523 10.1111/j.1743-6109.2006.00287.x

[84] Mamas MA, Reynard JM, Brading AF (2003). Nitric oxide and the lower urinary tract: Current concepts, future prospects. Urology, 61:1079-85. 12809865 10.1016/s0090-4295(03)00131-6

[85] Abdel-Hamid IA, El Naggar EA, El Gilany AH (2001). Assessment of as needed use of pharmacotherapy and the pause-squeeze technique in premature ejaculation. Int J Impot Res, 13:41-5. 11313839 10.1038/sj.ijir.3900630

[86] Chen J, Mabjeesh NJ, Matzkin H, Greenstein A (2003). Efficacy of sildenafil as adjuvant therapy to selective serotonin reuptake inhibitor in alleviating premature ejaculation. Urology, 61:197-200. 12559295 10.1016/s0090-4295(02)02075-7

[87] Salonia A, Maga T, Colombo R, Scattoni V, Briganti A, Cestari A, et al (2002). A prospective study comparing paroxetine alone versus paroxetine plus sildenafil in patients with premature ejaculation. J Urol, 168:2486-9. 12441946 10.1016/S0022-5347(05)64174-2

[88] Aversa A, Pili M, Francomano D, Bruzziches R, Spera E, La Pera G, et al (2009). Effects of vardenafil administration on intravaginal ejaculatory latency time in men with lifelong premature ejaculation. Int J Impot Res, 21:221-7. 19474796 10.1038/ijir.2009.21

[89] Mattos RM, Marmo Lucon A, Srougi M (2008). Tadalafil and fluoxetine in premature ejaculation: Prospective, randomized, double-blind, placebo-controlled study. Urol Int, 80: 162-5. 18362486 10.1159/000112607

[90] McMahon CG, Stuckey BG, Andersen M, Purvis K, Koppiker N, Haughie S, et al (2005). Efficacy of sildenafil citrate (Viagra) in men with premature ejaculation. J Sex Med, 2:368-75. 16422868 10.1111/j.1743-6109.2005.20351.x

[91] Althof SE, Rowland DL (2008). Identifying constructs and criteria for the diagnosis of premature ejaculation: Implication for making errors of classification. BJU Int, 102:708-12. 18522629 10.1111/j.1464-410X.2008.07790.x

[92] Sáenz de Tejada I, Anglin G, Knight JR, Emmick JT (2002). Effects of tadalafil on erectile dysfunction in men with diabetes. Diabetes Care, 25(12):2159-64. 12453954 10.2337/diacare.25.12.2159

[93] Liao X, Qiu S, Bao Y, Wang W, Yang L, Wei Q (2019). Comparative efficacy and safety of phosphodiesterase type 5 inhibitors for erectile dysfunction in diabetic men: a Bayesian network meta-analysis of randomized controlled trials. World J Urol, 37(6):1061-1074. 30523399 10.1007/s00345-018-2583-1

[94] Buvat J, van Ahlen H, Schmitt H, Chan M, Kuepfer C, Varanese L (2006). Efficacy and safety of two dosing regimens of tadalafil and patterns of sexual activity in men with diabetes mellitus and erectile dysfunction: Scheduled use vs. on-demand regimen evaluation (SURE) study in 14 European countries. J Sex Med, 3(3):512-20. 16681477 10.1111/j.1743-6109.2006.00249.x

[95] Ziegler D, Merfort F, Van Ahlen H, Yassin A, Reblin T, Neureither M (2006). Efficacy and safety of flexible-dose vardenafil in men with type 1 diabetes and erectile dysfunction. J Sex Med, 3(5):883-891. 16942532 10.1111/j.1743-6109.2006.00295.x

[96] García-Perdomo HA, Echeverría-García F, Tobías A (2017). Effectiveness of Phosphodiesterase 5 Inhibitors in the Treatment of Erectile Dysfunction in Patients with Spinal Cord Trauma: Systematic Review and Meta-Analysis. Urol Int, 98(2):198-204. 27509143 10.1159/000448290

[97] Soler JM, Previnaire JG, Denys P, Chartier-Kastler E (2007). Phosphodiesterase inhibitors in the treatment of erectile dysfunction in spinal cord-injured men. Spinal Cord, 45(2):169-73. 16801935 10.1038/sj.sc.3101950

[98] Khorrami MH, Javid A, Moshtaghi D, Nourimahdavi K, Mortazavi A, Zia HR (2010). Sildenafil efficacy in erectile dysfunction secondary to spinal cord injury depends on the level of cord injuries. Int J Androl, 33(6):861-4. 20050938 10.1111/j.1365-2605.2009.01033.x

[99] Del Popolo G, Li Marzi V, Mondaini N, Lombardi G (2004). Time/duration effectiveness of sildenafil versus tadalafil in the treatment of erectile dysfunction in male spinal cord-injured patients. Spinal Cord, 42(11):643-8. 15289800 10.1038/sj.sc.3101617

[100] Nehra A, Grantmyre J, Nadel A, Thibonnier M, Brock G (2005). Vardenafil improved patient satisfaction with erectile hardness, orgasmic function and sexual experience in men with erectile dysfunction following nerve sparing radical prostatectomy. J Urol, 173(6):2067-71. 15879836 10.1097/01.ju.0000158456.41788.93

[101] Limoncin E, Gravina GL, Corona G, Maggi M, Ciocca G, Lenzi A, et al (2017). Erectile function recovery in men treated with phosphodiesterase type 5 inhibitor administration after bilateral nerve-sparing radical prostatectomy: a systematic review of placebo-controlled randomized trials with trial sequential analysis. Andrology, 5(5):863-872. 28787547 10.1111/andr.12403

[102] Raina R, Lakin MM, Agarwal A, Mascha E, Montague DK, Klein E, et al (2004). Efficacy and factors associated with successful outcome of sildenafil citrate use for erectile dysfunction after radical prostatectomy. Urology, 63(5):960-6. 15134989 10.1016/j.urology.2003.12.012

[103] Fode M, Østergren PB, Jensen CFS, Jakobsen H, Sønksen J (2018). Treatment effects of phosphodiesterase-5 inhibitors may improve with time following nerve-sparing radical prostatectomy. Scand J Urol, 52(2):108-110. 29057723 10.1080/21681805.2017.1387603

[104] Miner MM, Barnes A, Janning S (2010). Efficacy of phosphodiesterase type 5 inhibitor treatment in men with erectile dysfunction and dyslipidemia: a post hoc analysis of the vardenafil statin study. J Sex Med, 7(5):1937-47. 20202105 10.1111/j.1743-6109.2010.01766.x

[105] Warde N (2011). Therapy: Two birds, one stone: Tadalafil is an effective treatment for men with both BPH-LUTS and ED. Nat Rev Urol, 8:643. 22083242 10.1038/nrurol.2011.165

[106] Shamloul R, Ghanem H (2013). Erectile dysfunction. Lancet, 381(9861):153-65. 23040455 10.1016/S0140-6736(12)60520-0

[107] Baumhäkel M, Schlimmer N, Kratz M, Hackett G, Jackson G, Böhm M (2011). Cardiovascular risk, drugs and erectile function--a systematic analysis. Int J Clin Pract, 65(3):289-98. 21314866 10.1111/j.1742-1241.2010.02563.x

[108] Aversa A, Francomano D, Lenzi A (2015). Does testosterone supplementation increase PDE5-inhibitor responses in difficult-to-treat erectile dysfunction patients. Expert Opin Pharmacother, 16(5): 625-8. 25643866 10.1517/14656566.2015.1011124

[109] Jannini EA, Isidori AM, Aversa A, Lenzi A, Althof SE (2013). Which is first? The controversial issue of precedence in the treatment of male sexual dysfunctions. J Sex Med, 10(10):2359-69. 24112352 10.1111/jsm.12315

[110] B uvat J, Montorsi F, Maggi M, Porst H, Kaipia A, Colson MH, et al (2011). Hypogonadal men nonresponders to the PDE5 inhibitor tadalafil benefit from normalization of testosterone levels with a 1% hydroalcoholic testosterone gel in the treatment of erectile dysfunction (TADTEST study). J Sex Med, 8(1):284-93. 20704642 10.1111/j.1743-6109.2010.01956.x

[111] Hwang TI, Chen HE, Tsai TF, Lin YC (2006). Combined use of androgen and sildenafil for hypogonadal patients unresponsive to sildenafil alone. Int J Impot Res, 18(4):400-4. 16395321 10.1038/sj.ijir.3901446

[112] Yassin DJ, Yassin AA, Hammerer PG (2014). Combined testosterone and vardenafil treatment for restoring erectile function in hypogonadal patients who failed to respond to testosterone therapy alone. J Sex Med 11(2):543-52. 24251448 10.1111/jsm.12378

[113] Yassin AA, Saad F, Diede HE (2006). Testosterone and erectile function in hypogonadal men unresponsive to tadalafil: results from an open-label uncontrolled study. Andrologia, 38(2):61-8. 16529577 10.1111/j.1439-0272.2006.00712.x

[114] Porst H, Burnett A, Brock G, Ghanem H, Giuliano F, Glina S, et al (2013). SOP conservative (medical and mechanical) treatment of erectile dysfunction. J Sex Med, 10(1):130-71. 23343170 10.1111/jsm.12023

[115] Yan H, Zong H, Cui Y, Li N, Zhang Y (2014). The efficacy of PDE5 inhibitors alone or in combination with alpha-blockers for the treatment of erectile dysfunction and lower urinary tract symptoms due to benign prostatic hyperplasia: a systematic review and meta-analysis. J Sex Med, 11(6):1539-45. 24621088 10.1111/jsm.12499

[116] El-Sisi AA, Hegazy SK, Salem KA, AbdElkawy KS (2013). Atorvastatin improves erectile dysfunction in patients initially irresponsive to Sildenafil by the activation of endothelial nitric oxide synthase. Int J Impot Res, 25(4):143-8. 23324897 10.1038/ijir.2012.46

[117] Dadkhah F, Safarinejad MR, Asgari MA, Hosseini SY, Lashay A, Amini E (2010). Atorvastatin improves the response to sildenafil in hypercholesterolemic men with erectile dysfunction not initially responsive to sildenafil. Int J Impot Res, 22(1):51-60. 19865092 10.1038/ijir.2009.48

[118] Herrmann HC, Levine LA, Macaluso J Jr, Walsh M, Bradbury D, Schwartz S, et al (2006). Can atorvastatin improve the response to sildenafil in men with erectile dysfunction not initially responsive to sildenafil? Hypothesis and pilot trial results. J Sex Med, 3(2):303-8. 10.1111/j.1743-6109.2005.00156.x 16490024

[119] Jaffe JS, Antell MR, Greenstein M, Ginsberg PC, Mydlo JH, Harkaway RC (2004). Use of intraurethral alprostadil in patients not responding to sildenafil citrate. Urology, 63(5):951-4. 15134987 10.1016/j.urology.2003.11.039

[120] Safarinejad MR (2006). Salvage of sildenafil failures with cabergoline: a randomized, double-blind, placebo-controlled study. Int J Impot Res, 18(6):550-8. 16625231 10.1038/sj.ijir.3901476

[121] Gutierrez P, Hernandez P, Mas M (2005). Combining programmed intracavernous PGE1 injections and sildenafil on demand to salvage sildenafil nonresponders. Int J Impot Res, 17(4):354-8. 15703770 10.1038/sj.ijir.3901290

[122] Verze P, Margreiter M, Esposito K, Montorsi P, Mulhall J (2015). The Link Between Cigarette Smoking and Erectile Dysfunction: A Systematic Review. Eur Urol Focus, 1(1):39-46. 28723353 10.1016/j.euf.2015.01.003

[123] Harte CB, Meston CM (2012). Association between smoking cessation and sexual health in men. BJU Int, 109:888-96. 21883852 10.1111/j.1464-410X.2011.10503.x PMC3235242

[124] Grover S, Mattoo SK, Pendharkar S, Kandappan V (2014). Sexual dysfunction in patients with alcohol and opioid dependence. Indian J Psychol Med, 36:355-65. 25336765 10.4103/0253-7176.140699 PMC4201785

[125] Bacon CG, Mittleman MA, Kawachi I, Giovannucci E, Glasser DB, Rimm EB (2003). Sexual function in men older than 50 years of age: results from the health professionals follow-up study. Ann Intern Med, 139:161-8. 12899583 10.7326/0003-4819-139-3-200308050-00005

[126] Kalter-Leibovici O, Wainstein J, Ziv A, Harman-Bohem I, Murad H, Raz I (2005). Clinical, socioeconomic, and lifestyle parameters associated with erectile dysfunction among diabetic men. Diabetes Care, 28:1739-44. 15983328 10.2337/diacare.28.7.1739

[127] Larsen SH, Wagner G, Heitmann BL (2007). Sexual function and obesity. Int J Obes (Lond), 31:1189-98. 17372616 10.1038/sj.ijo.0803604

[128] Kolotkin RL, Head S, Hamilton M, Tse CK (1995). Assessing Impact of Weight on Quality of Life. Obes Res, 3:49-56. 10.1002/j.1550-8528.1995.tb00120.x 7712359

[129] Esposito K, Giugliano F, Maiorino MI, Giugliano D (2010). Dietary factors, Mediterranean diet and erectile dysfunction. J Sex Med, 7:2338-45. 20487239 10.1111/j.1743-6109.2010.01842.x

[130] Nicolosi A, Moreira ED Jr, Shirai M, Bin Mohd Tambi MI, Glasser DB (2003). Epidemiology of erectile dysfunction in four countries: cross-national study of the prevalence and correlates of erectile dysfunction. Urology, 61:201-6. 12559296 10.1016/s0090-4295(02)02102-7

[131] Derby CA, Mohr BA, Goldstein I, Feldman HA, Johannes CB, McKinlay JB (2000). Modifiable risk factors and erectile dysfunction: can lifestyle changes modify risk? Urology, 56:302-6. 10925098 10.1016/s0090-4295(00)00614-2

[132] Cialis^®^ (tadalafil) (2018). Full Prescribing Information. Indianapolis, IN: Eli Lilly and Company.

